# Urinary Retention

**Published:** 1875-09

**Authors:** 


URINARY RETENTION.—*Boston Medical and Surgical Journal,* May 13, reports the following method as commonly resorted to at the Boston City Hospital in primary treatment of urinary retention: A boy, fourteen years old fell, receiving a contusion of the hip; retention of urine followed, which could not be relieved; notwithstanding repeated attempts with the catheter, the bladder was distended to the umbilicus. Dr. Ingalls punctured the bladder above the pubes with the aspirator, and drew off three pints of alkaline urine, with slight pain, great relief, and no ether. The next day patient was catheterized, afterwards micturated with ease and was well. This course Dr. I. finds admirable, as the urethra rests and recovers from conjestion, swelling, and tenderness. Generally one, two or three aspirations are required, relief certain, the pain slight, and the danger is nothing so far as is shown by the pretty large experience of this hospital. The operation may be repeated two or three times a day with safety.—*Charleston Medical Journal.*

